# The estrogen receptor influences microtubule-associated protein tau (MAPT) expression and the selective estrogen receptor inhibitor fulvestrant downregulates MAPT and increases the sensitivity to taxane in breast cancer cells

**DOI:** 10.1186/bcr2598

**Published:** 2010-06-28

**Authors:** Hirokuni Ikeda, Naruto Taira, Fumikata Hara, Takeo Fujita, Hiromasa Yamamoto, Junichi Soh, Shinichi Toyooka, Tomohiro Nogami, Tadahiko Shien, Hiroyoshi Doihara, Shinichiro Miyoshi

**Affiliations:** 1 Department of Cancer and Thoracic Surgery, Okayama University Graduate School of Medicine, Dentistry, and Pharmaceutical Sciences, 2-5-1 Shikata-cho, Okayama-city, Okayama, 700-8558, Japan; 2 Department of Breast and Endocrine Surgery, National Hospital Organization, National Shikoku Cancer Center, Matsuyama, Kou 160, Minami-umemoto-machi, Matsuyama-city, Ehime, 791-0280, Japan; 3 Division of Digestive Surgery, National Cancer Center Hospital East Kashiwa, 6-5-1 Kashiwanoha, Kashiwa-city, Chiba, 277-8577, Japan

## Abstract

**Introduction:**

Microtubule-associated protein tau (MAPT) inhibits the function of taxanes and high expression of MAPT decreases the sensitivity to taxanes. The relationship between estrogen receptor (ER) and MAPT in breast cancer is unclear. In this study, we examined the correlation of MAPT expression with the sensitivity of human breast cancer cells to taxanes, and the relationship between ER and MAPT.

**Methods:**

The correlation between MAPT expression and sensitivity to taxanes was investigated in 12 human breast cancer cell lines. Alterations in cellular sensitivity to taxanes were evaluated after knockdown of MAPT expression. ER expression was knocked down or stimulated in MAPT- and ER-positive cell lines to examine the relationship between ER and MAPT. The cells were also treated with hormone drugs (tamoxifen and fulvestrant) and taxanes.

**Results:**

mRNA expression of MAPT did not correlate with sensitivity to taxanes. However, expression of MAPT protein isoforms of less than 70 kDa was correlated with a low sensitivity to taxanes. Downregulation of MAPT increased cellular sensitivity to taxanes. MAPT protein expression was increased by stimulation with 17-β-estradiol or tamoxifen, but decreased by ER downregulation and by fulvestrant, an ER inhibitor. The combination of fulvestrant with taxanes had a synergistic effect, whereas tamoxifen and taxanes had an antagonistic effect.

**Conclusions:**

Expression of MAPT protein isoforms of less than 70 kDa is correlated with a low sensitivity to taxanes in breast cancer cells. ER influences MAPT expression and fulvestrant increases the sensitivity to taxanes in MAPT- and ER-positive breast cancer cells.

## Introduction

Taxanes are important drugs for treatment of breast cancer [1-3]. These drugs bind to tubulin and suppress spindle microtubule dynamics, which leads to cell cycle arrest in G2/M phase followed by apoptosis [4-6].

Several mechanisms of taxane resistance have been described, including overexpression of the drug efflux pump MDR-1/P-gp, HER-2 overexpression, tubulin mutation, and variable expression of tubulin isotypes and stathmin [4,7-12]. Microtubule-associated protein-tau (MAPT), which is implicated in the pathogenesis of Alzheimer's disease, is associated with another mechanism of taxane resistance. MAPT binds to both the outer and inner surfaces of microtubules, leading to tubulin assembly and microtubule stabilization. Since taxanes also bind to the inner surface of microtubules, MAPT obstructs the function of the drug [5,6,13,14]. Rouzier *et al*. found that low MAPT expression was associated with higher rates of a pathologic complete response to preoperative paclitaxel and 5-fluorouracil, doxorubicin, cyclophosphamide (paclitaxel/FAC) chemotherapy [5]. This group also showed that MAPT overexpression was correlated with resistance to paclitaxel and that knockdown of MAPT with small interfering RNA (siRNA) reversed the resistance to taxanes *in vitro *[5].

MAPT has six isoforms that are spliced from a single gene. These isoforms differ by having three or four conserved repeat motifs in the microtubule-binding domain and none, one or two insertions in the N-terminal projection domain. Isoforms with four C-terminal repeats have a higher affinity for microtubules than isoforms with three such repeats [13-17]. However, the function of each isoform is unknown.

Previous experimental studies have shown that MAPT expression is increased by estrogen *in vitro *and *in vivo *[18,19], and clinical studies have shown a positive correlation of MAPT levels with estrogen receptors (ER) expression [20,21]. Jonna *et al*. found that estrogen stimulation upregulated MAPT mRNA in MCF-7 cells in microarray analysis [22], and the MAPT gene is considered to contain an imperfect ER response element upstream of its promoter. The ER plays a key role in the development and progression of breast cancer, but it is unknown if ER stimulation induces MAPT expression in breast cancer cells.

Hormonal drugs play an important role in breast cancer therapy. The selective ER inhibitor, fulvestrant, inhibits estrogen signaling through the ER in two ways: by competing with estradiol binding to the ER, and by increasing the turnover of ER to decrease the ER protein level in breast cancer cells. In contrast, tamoxifen, a selective ER modulator, is an ER antagonist but often displays estrogen-like agonist activity [22-24]. Therefore, fulvestrant and tamoxifen may have different effects on MAPT expression via the ER.

Previous *in vitro *studies show that tamoxifen has an antagonistic effect on anti-cancer drugs [25,26]. Several clinical studies that used tamoxifen for hormone therapy have found that it has an antagonistic effect on chemotherapy drugs when it is used concurrently with them, and that the results of the combined use of tamoxifen with chemotherapy drugs is inferior, compared with using the drugs sequentially [27-30]. The effect of combination treatment using other modern hormone therapies, such as aromatase inhibitors or fulvestrant, has not been examined thoroughly.

In this study, we examined the relationship between the MAPT expression and the sensitivity to taxanes, the effect of ER expression or modulation on MAPT expression, and the combined impact of hormones and taxanes on anti-cancer activity and taxane resistance in breast cancer cell lines.

## Materials and methods

### Cell culture and agents

Twelve human breast cancer cell lines were used in the study: MCF-7, MDA-MB-231, SK-BR-3 and ZR75-1 were obtained from the American Type Culture Collection (Rockville, MD, USA); YMB1-E was kindly provided by the Tohoku University Institute of Development, Aging and Cancer Cell Resource Center for Biomedical Research; and MDA-MB-134-VI, HCC38, HCC1143, HCC1569, HCC1806, HCC1937 and HCC3153 were kindly provided by Adi F. Gazdar (Hamon Center for Therapeutic Oncology Research and Department of Pathology, University of Texas Southwestern Medical Center at Dallas, Dallas, TX, USA). Cells were maintained at 37°C in 5% CO_2 _in RPMI-1640 medium (Sigma-Aldrich, St. Louis, MO, USA) containing 10% heat-inactivated fetal bovine serum and 1% penicillin-streptomycin.

Paclitaxel, docetaxel, fulvestrant, 17-β estradiol and tamoxifen were purchased from Sigma-Aldrich. Vinorelbine and doxorubicin were obtained from Kyowa Hakkoh (Tokyo, Japan). Cells were cultured in a phenol-free medium containing 10% dextran-coated, charcoal-treated FCS (Thermo Scientific, Waltham, MA, USA) and then treated with the above agents alone or in combination.

### Small interfering RNA

Expression levels of MAPT and ER alpha were knocked down by transfection of the cells with two anti-MAPT siRNAs (5'-CGG GAC TGG AAG CGA TGA CAA-3' and 5'-CCG CCA GGA GTT CGA AGT GAT-3'; Qiagen, Valencia, CA, USA) and an anti-ER alpha siRNA (5'-GAG ACT TGA ATT AAT AAG TGA-3'; Qiagen), respectively. Scrambled siRNA (AllStars Negative Control siRNA, Qiagen) was used as the control. Transfection of siRNA was performed using HiPerfect Transfection Reagent (Qiagen) according to the manufacturer's protocol.

### mRNA and protein expression analysis

Total RNA was extracted from cell pellets and cDNA was synthesized using a High Capacity cDNA Reverse Transcription Kit (Applied Biosystems, Foster City, CA, USA) in accordance with the manufacturer's protocol. Quantitative real-time PCR was performed using the Step One™ Real-Time PCR System (Applied Biosystems) with 18S rRNA as an internal control (Applied Biosystems). The sequences of primers and the probe were as follows: MAPT, forward primer: 5'-TAG GCA ACA TCC ATC ATA AAC CA-3'; reverse primer: 5'-TCG ACT GGA CTC TGT CCT TGA A-3'; and the FAM-TAMR probe: 5'-TGG CCA GGT GGA AG-3' (Invitrogen, Carlsbad, CA, USA). Data were analyzed using the relative standard curve method.

Samples from cultured cells were prepared for Western blot analysis, as previously described [31]. The samples were separated on a NuPAGE Bis-Tris Gel 4 to 12% (Invitrogen) and electroblotted onto a polyvinylidene fluoride membrane. Primary antibodies for Western blotting were as follows: MAPT (T1029, United States Biological, Swampscott, MA, USA) [5,6]; ERα (Santa Cruz Biotechnology, Santa Cruz, CA, USA); and actin (Sigma-Aldrich). Blots were exposed to a horseradish peroxidase-conjugated secondary antibody (Santa Cruz Biotechnology) with development using enhanced chemiluminescence detection (ECL Kit, Amersham Pharmacia Biotech, Chandler, AZ, USA).

### Effects of agents on cells

A cell viability assay was performed as previously described [31], in which IC_50 _values were determined for the anti-proliferative activity of each drug. Experiments were performed independently four times and the data shown are the average of the four assays. The combination effect of two agents was evaluated using the Combination Index (C.I), which was calculated using Calcusyn software (Biosoft, Cambridge, UK). The definition of C.I is as follows: C.I = (D)1/(Dx)1 + (D)2/(Dx)2 + (D)1(D)2/(Dx)1(Dx)2, where (Dx)1 is the dose of Drug 1 alone required to produce an X% effect; (D)1 is the dose of Drug 1 required to produce the same X% effect in combination with Drug 2; (Dx)2 is the dose of Drug 2 alone required to produce an X% effect; and (D)2 is the dose of Drug 2 required to produce the same X% effect in combination with Drug 1. C.I < 1, 1 and > 1 indicates a synergistic effect, an additive effect, and an antagonistic effect, respectively. Cell cycle effects were examined by flow cytometry as previously described [32,33].

### Immunofluorescence

Cells were fixed in 4% paraformaldehyde, washed with cold PBS, and incubated in PBS containing 0.1% Triton X-100. After permeabilization, the cells were incubated in blocking buffer (PBS containing 0.1% Tween-20 and 3% BSA) containing antibodies against α-tubulin (Sigma-Aldrich). After washing with 0.1% Tween PBS, the cells were incubated in blocking buffer containing an anti-mouse AlexaFluor 488-conjugated secondary antibody (green) (Invitrogen). After washing again with 0.1% Tween PBS, the cells were incubated in PBS containing DAPI (blue) (Invitrogen). Immunofluorescence microscopy was performed using Biozero (Keyence, Osaka, Japan).

## Results

### MAPT expression and drug sensitivity

**Figure 1:**
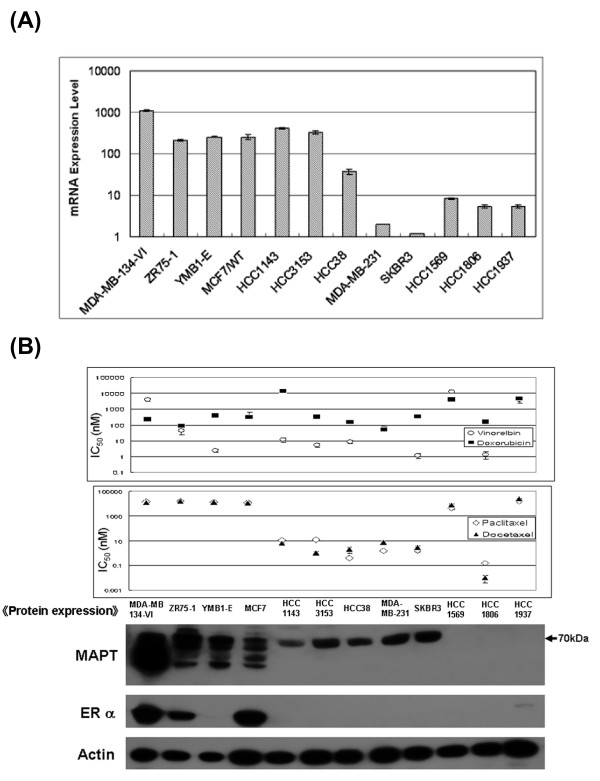
**MAPT expression and drug sensitivity**. **A: MAPT **mRNA expression in human breast cancer cell lines. PCR was used to assess MAPT mRNA levels in 12 human breast cancer cell lines. Since MAPT expression was lowest in SK-BR-3 cells, this expression level was taken as the standard for comparison with other cell lines. Cell line ZR75-1 has a naturally high MAPT expression [5,6]. Other cell lines with an mRNA expression higher than that of cell line ZR75-1 were classified as high mRNA expression cell lines. Real-time PCR revealed six cell lines in this category: MDA-MB-134-VI, ZR75-1, YMB1-E, MCF-7, HCC1143, and HCC3153. **B: **The correlation between MAPT protein expression and sensitivity to anti-cancer drugs. Western blot analysis shows that four cell lines (MDA-MB-134-VI, ZR75-1, YMB1-E, and MCF-7) have multiple protein bands ranging from 50 to 70 kDa. Cell lines HCC1143 and HCC3153 each show only one band at around 70 kDa, despite having a high MAPT mRNA expression. Cell lines HCC38, MDA-MB-231 and SK-BR-3 have low MAPT mRNA expression, but show one band at around 70 kDa. MTS assays were used to assess the sensitivity to paclitaxel, docetaxel, vinorelbine, and doxorubicin, and to determine the IC_50 _values for drug sensitivity. The upper figure shows the sensitivity to vinorelbine and doxorubicin, and the lower figure shows the sensitivity to paclitaxel and docetaxel. Four cell lines with multiple protein bands show a low sensitivity to taxanes, and five cell lines with only one band at around 70 kDa show a high sensitivity to taxanes. This trend was not observed for vinorelbine and doxorubicin. Cell lines HCC1569 and HCC1937, both having a low MAPT expression, show a low sensitivity to all four anti-cancer drugs. Cell line HCC1937 was the BRCA1-defective breast cancer cell line. The characteristics of HCC1569 are unclear.

MAPT mRNA expression was assessed using quantitative real-time PCR and MAPT protein expression was assessed using Western blot analysis in 12 human breast cancer cell lines (Figure 1A, B). Six cell lines showed high MAPT mRNA expression. Four of the six cell lines showed multiple protein bands of 50 to 70 kDa, and the other two cell lines showed only one band at around 70 kDa. Three cell lines with low MAPT mRNA expression also showed MAPT protein expression at around 70 kDa.

To analyze the correlation between MAPT expression and drug sensitivity, MTS assays were performed to determine the IC_50 _values for the sensitivity of the 12 cell lines to paclitaxel, docetaxel, vinorelbine, and doxorubicin. Four of the six cell lines that showed high MAPT mRNA expression had a low sensitivity to taxanes, while the remaining two cell lines showed a high sensitivity. The five cell lines expressing only the MAPT protein isoform at around 70 kDa had a high sensitivity to taxanes, but all four cell lines with MAPT protein isoforms of less than 70 kDa showed a low sensitivity to taxanes. This trend was not observed for vinorelbine and doxorubicin. Cell line HCC1937, which has a low MAPT expression, showed a low sensitivity to all four anti-cancer drugs. HCC1937 is the BRCA1-defective breast cancer cell line and has a low sensitivity to paclitaxel and doxorubicin [34]. Cell line HCC1569, which has a low MAPT expression, showed a low sensitivity to all four anti-cancer drugs, but its character is unclear. Our results suggest that MAPT mRNA expression is insufficient as a predictor of taxane sensitivity; that the MAPT protein isoform at around 70 kDa does not cause a low sensitivity to taxanes; and that MAPT protein isoforms of less than 70 kDa are most correlated with a low sensitivity to taxanes.

### Downregulation of MAPT expression and alteration of cellular sensitivity to taxanes

**Figure 2:**
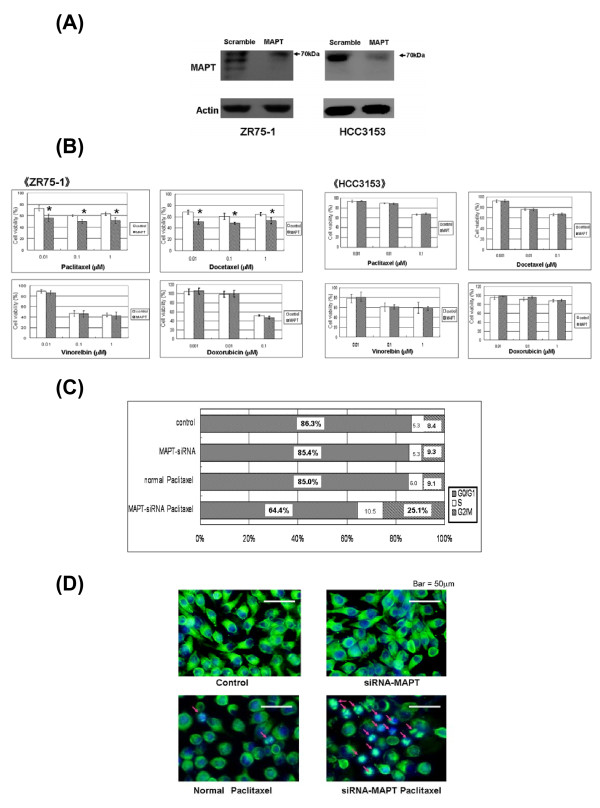
**Downregulation of MAPT expression and alteration of cellular sensitivity to taxanes**. **A**: Downregulation of MAPT expression with siRNA. siRNA was used to knock down MAPT expression in ZR75-1 and HCC3153 cells. Cells were harvested 72 hours after transfection for Western blot analysis. **B: **Cell viability determined by an MTS assay after the knockdown of MAPT. Cells were seeded 24 hr after transfection on a 96-well plate at 5 × 10^3 ^cells/well and incubated for 24 hr. The cells were then cultivated for 72 hr in the presence of various concentrations of drugs. After this treatment, four independent MTS assays were performed. The data shown are the average of these four assays. In ZR75-1 cells knockdown of MAPT significantly increased sensitivity to taxanes, but did not alter sensitivity to vinorelbine or doxorubicin. In HCC3153 cells knockdown of MAPT did not alter sensitivity to taxanes. *P < 0.05, indicates a significant difference, compared with the control (unpaired Student's test). **C: **Analysis of the cell cycle using flow cytometry. At 24 hr after transfection, cells were exposed to low-dose paclitaxel (25 nM) for 72 hr. Afterwards, a cell cycle analysis was performed using flow cytometry. The percentage of cells in the G2/M phase was higher in cells that had been exposed to low-dose taxanes after MAPT knockdown, compared with the controls. (The data show ZR75-1 cells with paclitaxel.) **D: **Analysis of cell cycle and cell proliferation using immunofluorescence. After transfection, cells were seeded on six-well plates and incubated for 24 hr. After this treatment, the cells were exposed to low-dose paclitaxel (25 nM) for 24 hr. Immunofluorescence with an anti-α-tubulin antibody was then performed. Paclitaxel caused an increase in apoptotic cells from 14/100 cells (that is, the control cells) to 29/100 cells (that is, MAPT knockdown cells). There was also repressed proliferation, compared with the controls. (The arrows indicate apoptotic ZR75-1 cells.)

To examine the role of MAPT protein in taxane resistance, siRNA was used to knock down MAPT expression in MAPT-positive cell lines as confirmed by Western blot analysis. ZR75-1, MCF-7 and HCC3153 cells were used in our study. The level of siRNA knockdown was approximately 65%. MDA-MB-134-VI, YMB1-E and HCC1143 cells could not be knocked down MAPT expression successfully. After MAPT knockdown, the cells were cultured with various concentrations of drugs and MTS assays were performed. ZR75-1, MCF-7 cells has MAPT protein isoforms of less than 70 kDa, all isoforms were knocked down by siRNA, and knockdown of MAPT increased the sensitivity to taxanes in both cell lines, but did not alter the sensitivity to vinorelbine and doxorubicin. In contrast, in HCC3153 cells, in which only the MAPT isoform at around 70 kDa is expressed, siRNA knockdown of this isoform did not change the sensitivity to taxanes (Figure 2A, B). Based on these results, we suggest that MAPT protein isoforms of less than 70 kDa largely determine the sensitivity to taxanes.

Taxanes bind to tubulin and suppress spindle microtubule dynamics, which leads to arrest of the cell cycle in G2/M phase. We hypothesized that MAPT expression, and especially MAPT protein isoforms of less than 70 kDa, might block this activity of the drug. Using flow cytometry and immunofluorescence, changes in the cell cycle and cell proliferation were analyzed after knockdown of MAPT and exposure of the cells to low doses of taxanes. The percentage of cells in G2/M phase was higher in those exposed to low doses of taxanes after MAPT knockdown than in controls (Figure 2C). An increased percentage of sub-G1 phase cells was also noted for cells exposed to taxanes after MAPT knockdown (data not shown). Immunofluorescence with an anti-α-tubulin antibody (Figure 2D) showed that taxanes increased the amount of apoptotic cells and repressed cell proliferation after MAPT knockdown, in comparison with controls.

### The relationship between ER and MAPT expression

**Figure 3:**
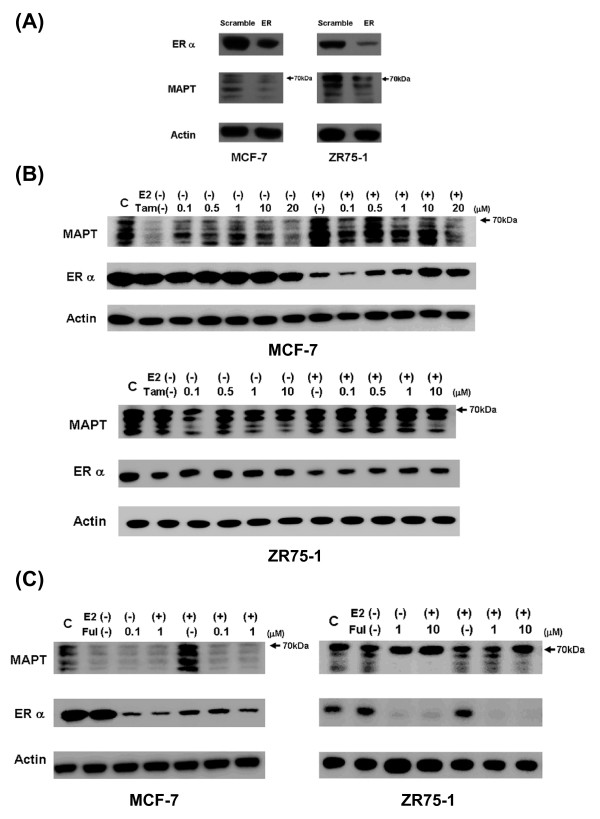
**The relationship between ER and MAPT expression. A**: Downregulation of ER expression with siRNA. siRNA was used to knock down ER expression in MCF-7 and ZR75-1 cells. Cells were harvested 72 hours after transfection for Western blot analysis. The MAPT protein expression decreased after ER knockdown in both cells lines. **B: **Treatment with 17-β estradiol and tamoxifen. Cells were seeded in serum-free medium and incubated. After incubation for 24 hr, cells were cultivated in a medium containing 17-β estradiol alone, tamoxifen alone, or various combinations of these two agents for 72 hr. They were then harvested for Western blot analysis. In MCF7 cells, MAPT expression was increased by 17-β estradiol. In the absence of 17-β estradiol, tamoxifen increased MAPT expression. This effect was highest at concentrations of 500 nM and 1 μM, and decreased at higher concentrations. With 17-β estradiol, tamoxifen at low concentrations decreased MAPT expression, but a high stimulatory effect was found at 500 nM. This effect decreased at high concentrations. In ZR75-1 cells, these changes were observed for protein isoforms of less than 70 kDa. MCF-7 cells were exposed to 1.0 nM 17-β estradiol and ZR75-1 cells to 10 nM 17-β estradiol. E2: 17-β estradiol; Tam: tamoxifen. **C: **Stimulation with 17-β estradiol and fulvestrant. Cells were seeded in a serum-free medium and incubated. After a 24-hr incubation, cells were cultivated in a medium containing 17-β estradiol alone, fulvestrant alone, or various combinations of the two agents for 72 hr. They were then harvested for Western blot analysis. Fulvestrant decreased ER and MAPT protein expression in both cell lines. These changes were more noticeable for protein isoforms of less than 70 kDa in ZR75-1 cells. MCF-7 cells were exposed to 1.0 nM 17-β estradiol and ZR75-1 cells to 10 nM 17-β estradiol. E2, 17-β estradiol; Ful, fulvestrant.

Three of the 12 cell lines were ER-positive and all three had expression of MAPT protein isoforms of less than 70 kDa (Figure 1A). To examine the relationship between ER and MAPT expression, siRNA was used to knock down ER expression in the MAPT- and ER-positive cell lines (MCF-7 and ZR75-1), as confirmed by Western blot analysis (The level of siRNA knockdown was approximately 85%). MAPT protein expression decreased after ER knockdown in both cells lines (Figure 3A). These results indicate that the MAPT level is influenced by ER in breast cancer cell lines. To examine this relationship in more detail, cells were stimulated with 17-β estradiol and treated with tamoxifen and fulvestrant. The cells were seeded in a serum-free medium, incubated for 24 hr, and then cultivated in a medium containing 17-β estradiol alone; tamoxifen alone; fulvestrant alone; or various combinations of these agents. Subsequent Western blot analysis was performed. In MCF7 cells, MAPT expression was increased by 17-β estradiol. In the absence of 17-β estradiol, exposure to tamoxifen increased MAPT expression. This effect was strongest at low concentrations of 500 nM to 1 μM. With 17-β estradiol, MAPT expression was decreased by tamoxifen at low concentrations, but increased by tamoxifen at 500 nM. The effect of increased MAPT expression was reduced at higher concentrations of tamoxifen. In ZR75-1 cells, these changes were observed for protein isoforms of less than 70 kDa (Figure 3B). Exposure to fulvestrant decreased ER and MAPT expression regardless of the drug concentration. In ZR75-1 cells, similar changes were observed for MAPT protein isoforms of less than 70 kDa (Figure 3C). These findings suggest that the ER modulates MAPT expression, especially for MAPT isoforms of less than 70 kDa. Tamoxifen stimulates the ER in the presence of 17-β estradiol, and significantly increases MAPT expression at low concentration, whereas fulvestrant decreases ER and MAPT expression at all concentrations.

### Combination treatment with hormone drugs and taxanes

**Figure 4:**
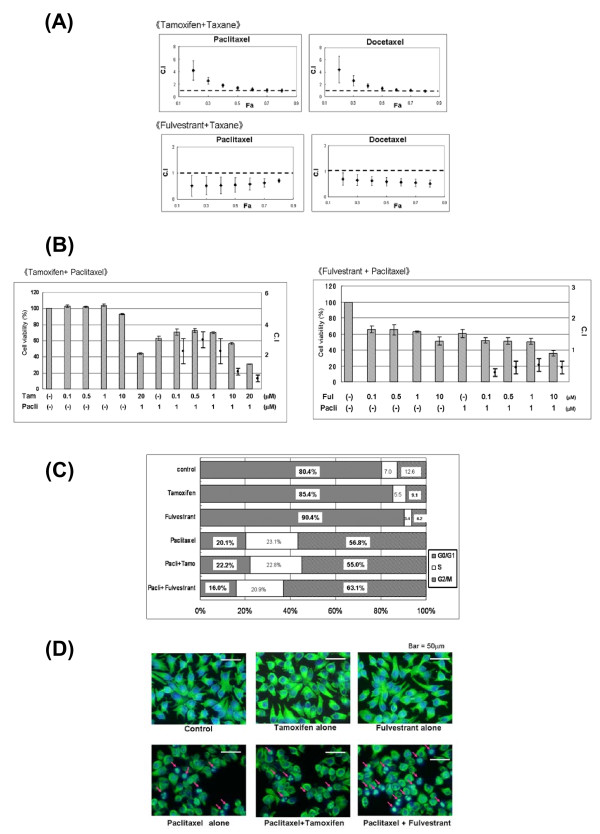
**Combination treatment with hormone drugs and taxanes. A**: Effect of combination treatment with hormone drugs and taxanes using a constant ratio design. The combination effect was evaluated using the Combination Index (C.I). Combination treatment with fulvestrant and taxanes showed a synergistic effect. Combination treatment with tamoxifen and taxanes had an antagonistic effect at low doses, but this effect became additive in both cell lines as the tamoxifen dose increased. (Data for MCF-7 cells are shown.) C.I, Combination Index; Fa, fraction affected. **B: **Effect of combination treatment with hormone drugs and taxanes using a non-constant ratio design. Cells were cultivated with 1 μM paclitaxel alone, with tamoxifen at various concentrations, with fulvestrant alone at various concentrations, and with combinations of these agents. The combination effect was evaluated using the Combination Index (C.I). In combination treatment with tamoxifen and paclitaxel, an antagonistic effect was observed at low concentrations, while an additive effect was found at high concentrations. The strongest antagonistic effect was observed at 500 nM. Combinations of fulvestrant and taxanes gave a synergistic effect. (Data are shown for MCF-7 cells with paclitaxel.) Pacli, paclitaxel; Tam, tamoxifen; Ful, fulvestrant; C.I, Combination Index. **C: **Evaluation of combination treatment by flow cytometry. Cells were cultivated with 50 nM paclitaxel alone, 100 nM tamoxifen alone, 75 nM fulvestrant alone, or with combinations of these agents. Combination treatment with fulvestrant and paclitaxel increased the percentage of cells in G2/M phase, compared with paclitaxel alone or combination treatment with paclitaxel and tamoxifen. (Data are shown for MCF-7 cells with paclitaxel.) **D: **Evaluation of combination treatment using immunofluorescence. Cells were cultivated with 50 nM paclitaxel alone, 100 nM tamoxifen alone, 75 nM fulvestrant alone, or combinations of these agents. An increase in apoptotic cells and repression of cell proliferation occurred in the combination treatment with fulvestrant and paclitaxel, compared with paclitaxel alone. These changes did not occur in combination treatment with tamoxifen. Apoptotic cells occurred in 11/100 cells with paclitaxel treatment alone; in 12/100 cells in combination treatment using tamoxifen; and in 25/100 cells in combination treatment using fulvestrant. (Arrows indicate apoptotic MCF-7 cells.)

Based on the above results, we hypothesized that a combination of hormone drugs and taxanes would increase anti-cancer activity and the sensitivity to taxanes. This hypothesis was tested in the MAPT- and ER-positive cell lines (MCF-7 and ZR75-1), with evaluation of the combination treatment using the Combination Index. The molar ratios for combinations of paclitaxel, docetaxel, tamoxifen and fulvestrant were determined based on the individual IC_50 _values (Table 1). This study was performed using a constant ratio design. The combination treatment with tamoxifen and taxanes had an antagonistic effect at a low dose, but this effect became additive in both cell lines as the dose of tamoxifen increased. In contrast, the combination of fulvestrant with taxanes showed a synergistic effect (Figure 4A, Table 2).

**Table 1:** Combination molar ratios for taxanes, tamoxifen and fulvestrant

Drugs	MCF-7	ZR75-1
	Ratio	Ratio
Tamoxifen : Paclitaxel	5:3	4:3
Tamoxifen : Docetaxel	5:3	4:3
Fulvestrant : Paclitaxel	5:4	1:1
Fulvestrant : Docetaxel	5:4	1:1

**Table 2:** Combination Index of hormone drugs and taxanes

	Tamoxifen			Fulvestrant		
	**Combination Index Values at**			**Combination Index Values at**		
**Cell line**	**ED50 (Average ± SD)**	**ED75 (Average ± SD)**	**r**	**ED50 (Average ± SD)**	**ED75 (Average ± SD)**	**r**

**(Paclitaxel)**						
MCF7	1.38(0.26)	1.00(0.28)	0.97	0.55(0.28)	0.66(0.12)	0.97
ZR75-1	1.42(0.35)	1.04(0.09)	0.98	0.45(0.22)	0.56(0.32)	0.97
**(Docetaxel)**						
MCF7	1.35(0.11)	0.91(0.16)	0.97	0.59(0.17)	0.53(0.14)	0.98
ZR75-1	1.33(0.37)	1.11(0.27)	0.97	0.53(0.31)	0.60(0.20)	0.98

The CI values at ED50 and ED 75 in the MCF-7 and ZR 75-1 cell lines. The values are the average of four independent experiments, with the SD given in parentheses. Combination treatment with fulvestrant and taxanes showed a stronger synergistic effect than the combination treatment with tamoxifen and taxanes in both cell lines. C.I, Combination Index; SD, standard deviation.

Since the effect of tamoxifen on MAPT expression differs depending on its concentration, the effect of concomitant use with taxanes may also differ depending on tamoxifen concentration. Thus, we performed combination treatment with tamoxifen at various concentrations, with the taxane concentration maintained at a certain level, and evaluated the results using the Combination Index (Figure 4B). This study was performed using a non-constant ratio design. The combination treatment with tamoxifen and taxanes gave an antagonistic effect at low concentrations, but an additive effect at high concentrations. The strongest antagonistic effect was found at 500 nM, and the differences in concomitant treatment effects caused by the tamoxifen concentration were similar to those for MAPT expression. Combination treatment with fulvestrant and taxanes gave a synergistic effect regardless of the fulvestrant concentration.

The effects of a combination of hormone drugs and taxanes on the cell cycle and cell proliferation were evaluated with flow cytometry and immunofluorescence. Treatment with tamoxifen alone or fulvestrant alone increased the percentage of cells in G1 phase and taxanes increased the percentage of cells in G2/M phase, compared with controls. The combination treatment with tamoxifen and taxanes increased the percentage of cells in G1 phase and decreased the percentage in G2/M phase slightly, compared with the respective values with each taxane alone. The combination of fulvestrant and taxanes did not alter the percentage of cells in G1 phase, despite the presence of fulvestrant, but the number of cells in G2/M phase increased compared with each taxane alone (Figure 4C). We also found that the percentage of sub-G1 phase cells increased after the combination treatment with fulvestrant and taxanes, compared to treatment with each taxane alone or the combination of tamoxifen with taxanes (data not shown).

Immunofluorescence showed that an increase in apoptotic cells and repression of cell proliferation occurred in the combination treatment with fulvestrant and paclitaxel, compared with paclitaxel alone. These changes did not occur in the combination treatment with tamoxifen (Figure 4D). These results indicate that fulvestrant supports taxane drug function and has a synergistic effect with taxanes.

## Discussion

In this study, we obtained several new findings on MAPT expression and the effects of taxanes. First, the sensitivity to taxanes is influenced by MAPT protein isoforms of less than 70 kDa. MAPT has six isoforms that give bands representing molecules ranging in size from 50 to 70 kDa, and MAPT protein isoforms have a significant impact on taxane sensitivity since they have different affinities for microtubules and different antagonistic effects on taxane [13-16]. Rouzier *et al*. provided the first report of the correlation of MAPT expression with the remission rate in subjects who received perioperative chemotherapy with a regimen including paclitaxel, and identified MAPT as a predictor of sensitivity to taxanes [5]. However, subsequent studies did not support the utility of MAPT as a predictor of the effect of taxanes [20,21,35,36]. Recently, Pusztai *et al*. performed a large-scale phase III clinical trial to compare doxorubicin and cyclophosphamide (AC) and AC followed by four courses of paclitaxel as adjuvant chemotherapy after surgery for breast cancer [36]. The prognosis of patients with MAPT expression was better than that of patients with no MAPT expression, although the utility of MAPT as a predictor of the taxane effect was not shown [36]. In clinical studies, RT-PCR and immunostaining are used for analysis of MAPT expression. The results of our *in vitro *study indicated that expression of MAPT protein isoforms less than 70 KDa had the most influence on sensitivity to taxanes. A discrepancy between MAPT mRNA expression and protein expression has been found previously [37], and thus analysis of MAPT mRNA expression may not be appropriate for examining the utility of MAPT as a predictor of taxane sensitivity. Furthermore, the status of the expression of different MAPT protein isoforms is important in determining sensitivity to taxanes, but immunohistochemistry cannot be used to evaluate each isoform. MAPT isoform expression in breast cancer tissues must be examined in detail to determine the exact correlation between MAPT expression and response to taxanes.

The second finding in the study involved clarification of the effect of the ER on the MAPT protein level in breast cancer cells. Clinical studies have suggested that MAPT expression has a positive correlation with ER expression and is influenced by ER signaling [20,21]. In our study in ER-positive and MAPT-positive breast cancer cell lines, expression of MAPT protein isoforms of less than 70 kDa, which have a large impact on sensitivity to taxanes, was affected by ER signaling. Furthermore, treatment of MAPT- and ER-positive cells with tamoxifen or fulvestrant had different effects on MAPT expression via the ER, which suggests that these drugs can alter cellular sensitivity to taxanes. In clinical treatment for breast cancer, the advantages and disadvantages of concomitant use of chemotherapeutic drugs and endocrine therapy have long been discussed. Several clinical studies of tamoxifen as hormone therapy have found that an antagonistic effect on concurrent chemotherapeutic agents, and that the results of giving tamoxifen concurrently with these agents are inferior to that of sequential administration [27-30]. These results suggest that concomitant chemotherapy and endocrine therapy should be avoided clinically. However, the effect of combination treatment using other modern hormone therapies, such as aromatase inhibitors or fulvestrant, has not been examined thoroughly.

Our third finding supports and complements the current idea on concomitant use of chemotherapy and endocrine therapy, and indicated a new possibility for concomitant use. Tamoxifen is an ER antagonist that also has estrogen-like agonist activity. It has been used as hormone drug for long-term therapy, but its effect are complicated and incompletely understood. Our results suggested that the effect of tamoxifen on ER signaling differs depending on the dose. MAPT protein expression was increased at low concentrations of tamoxifen of 500 nM - 1 μM, but decreased at higher concentrations. Several factors associated with resistance to chemotherapy via regulation by ER signaling have been identified [4,22,38-40]. Tamoxifen is thought to exert an antagonistic effect in concomitant use with chemotherapeutic drugs by increasing the expression of these factors via an agonistic effect on the ER. Active metabolites of tamoxifen also have different functions compared with the parent drug [41-43], and more detailed studies are needed to determine how tamoxifen and its metabolites influence chemotherapy *in vivo *and *in vitro*.

Fulvestrant decreased ER and MAPT expression at all concentrations. An MTS assay, flow cytometry, and immunofluorescence all showed that the combination of fulvestrant and taxanes had a synergistic effect, consistent with the finding of Sui *et al*. that fulvestrant combined with paclitaxel was effective in breast cancer cells *in vitro *[24]. Fulvestrant assists taxane function by downregulating the ER and ER-regulated factors associated with taxane resistance, and the combination of fulvestrant with taxanes increases the sensitivity of MAPT- and ER-positive breast cancer cells to taxanes.

ER-positive breast cancers clinically show a lower sensitivity to chemotherapy than do ER-negative breast cancers. This may be caused by the ER itself or by ER modulation of factors that result in resistance to chemotherapy. Our study indicates that the combination of modern hormone therapy with modern chemotherapy may become an effective therapy to ER-positive breast cancers.

## Conclusions

Expression of MAPT protein isoforms of less than 70 kDa is correlated with a low sensitivity to taxanes in breast cancer cells. ER influences MAPT expression and the selective ER inhibitor fulvestrant downregulates MAPT expression and increases the sensitivity to taxanes in MAPT- and ER-positive breast cancer cells.

## Abbreviations

C.I: Combination Index; ER: estrogen receptor; MAPT: microtubule-associated protein tau; siRNA: small interfering RNA.

## Competing interests

The authors declare that they have no competing interests.

## Authors' contributions

NT, FH, TF, TS and HD designed this study. HI wrote the manuscript with NT. HI and TN performed the experiments. HY and JS analyzed the data. ST and SM gave expert advice throughout the study.

## References

[B1] Piccart-Gebhart MJ Burzykowski T Buyse M Sledge G Carmichael J Lück HJ Mackey JR Nabholtz JM Paridaens R Biganzoli L Jassem J Bontenbal M Bonneterre J Chan S Basaran GA Therasse P Taxanes alone or in combination with anthracyclines as first-line therapy of patients with metastatic breast cancer J Clin Oncol 2008 26 1980 1986 10.1200/JCO.2007.10.8399 18421049

[B2] Cuppone F Bria E Carlini P Milella M Felici A Sperduti I Nisticò C Terzoli E Cognetti F Giannarelli D Taxanes as primary chemotherapy for early breast cancer: meta-analysis of randomized trials Cancer 2008 113 238 246 10.1002/cncr.23544 18470908

[B3] Mazouni C Kau SW Frye D Andre F Kuerer HM Buchholz TA Symmans WF Anderson K Hess KR Gonzalez-Angulo AM Hortobagyi GN Buzdar AU Pusztai L Inclusion of taxanes, particularly weekly paclitaxel, in preoperative chemotherapy improves pathologic complete response rate in estrogen receptor-positive breast cancers Ann Oncol 2007 18 874 880 10.1093/annonc/mdm008 17293601

[B4] McGrogan BT Gilmartin B Carney DN McCann A Taxanes, microtubules and chemoresistant breast cancer Biochim Biophys Acta 2008 1785 96 132 18068131 10.1016/j.bbcan.2007.10.004

[B5] Rouzier R Rajan R Wagner P Hess KR Gold DL Stec J Ayers M Ross JS Zhang P Buchholz TA Kuerer H Green M Arun B Hortobagyi GN Symmans WF Pusztai L Microtubule-associated protein tau: a marker of paclitaxel sensitivity in breast cancer Proc Natl Acad Sci USA 2005 102 8315 8320 10.1073/pnas.0408974102 15914550 PMC1149405

[B6] Wagner P Wang B Clark E Lee H Rouzier R Pusztai L Microtubule Associated Protein (MAP)-Tau: a novel mediator of paclitaxel sensitivity *in vitro *and *in vivo* Cell Cycle 2005 4 1149 1152 16103753 10.4161/cc.4.9.2038

[B7] Leonessa F Clarke R ATP binding cassette transporters and drug resistance in breast cancer Endocr Relat Cancer 2003 10 43 73 10.1677/erc.0.0100043 12653670

[B8] Yu D Jing T Liu B Yao J Tan M McDonnell TJ Hung MC Overexpression of ErbB2 blocks Taxol-induced apoptosis by upregulation of p21Cip1, which inhibits p34Cdc2 kinase Mol Cell 1998 2 581 591 10.1016/S1097-2765(00)80157-4 9844631

[B9] Berrieman HK Lind MJ Cawkwell L Do beta-tubulin mutations have a role in resistance to chemotherapy? Lancet Oncol 2004 5 158 164 10.1016/S1470-2045(04)01411-1 15003198

[B10] Shalli K Brown I Heys SD Schofield AC Alterations of beta-tubulin isotypes in breast cancer cells resistant to docetaxel FASEB J 2005 19 1299 1301 15946994 10.1096/fj.04-3178fje

[B11] Burkhart CA Kavallaris M Band Horwitz S The role of beta-tubulin isotypes in resistance to antimitotic drugs Biochim Biophys Acta 2001 1471 O1 O9 10.1016/s0304-419x(00)00022-6 11342188

[B12] Alli E Bash-Babula J Yang JM Hait WN Effect of stathmin on the sensitivity to antimicrotubule drugs in human breast cancer Cancer Res 2002 62 6864 6869 12460900

[B13] Kar S Fan J Smith MJ Goedert M Amos LA Repeat motifs of tau bind to the insides of microtubules in the absence of taxol EMBO J 2003 22 70 77 10.1093/emboj/cdg001 12505985 PMC140040

[B14] Smith CJ Anderton BH Davis DR Gallo JM Tau isoform expression and phosphorylation state during differentiation of cultured neuronal cells FEBS Lett 1995 375 243 248 10.1016/0014-5793(95)01221-Y 7498509

[B15] Gallo JM Hanger DP Twist EC Kosik KS Anderton BH: Expression and phosphorylation of a three-repeat isoform of tau in transfected non-neuronal cells Biochem J 1992 286 399 404 1530572 10.1042/bj2860399 PMC1132912

[B16] Goedert M Jakes R Expression of separate isoforms of human tau protein: correlation with the tau pattern in brain and effects on tubulin polymerization EMBO J 1990 9 4225 4230 2124967 10.1002/j.1460-2075.1990.tb07870.x PMC552204

[B17] Goode BL Chau M Denis PE Feinstein SC Structural and functional differences between 3-repeat and 4-repeat tau isoforms. Implications for normal tau function and the onset of neurodegenerative disease J Biol Chem 2000 275 38182 38189 10.1074/jbc.M007489200 10984497

[B18] Matsuno A Takekoshi S Sanno N Utsunomiya H Ohsugi Y Saito N Kanemitsu H Tamura A Nagashima T Osamura RY Watanabe K Modulation of protein kinases and microtubule-associated proteins and changes in ultrastructure in female rat pituitary cells: effects of estrogen and bromocriptine J Histochem Cytochem 1997 45 805 813 9199666 10.1177/002215549704500605

[B19] Ferreira A Caceres A Estrogen-enhanced neurite growth: evidence for a selective induction of Tau and stable microtubules J Neurosci 1991 11 392 400 1899446 10.1523/JNEUROSCI.11-02-00392.1991 PMC6575216

[B20] Andre F Hatzis C Anderson K Sotiriou C Mazouni C Mejia J Wang B Hortobagyi GN Symmans WF Pusztai L Microtubule-associated protein-tau is a bifunctional predictor of endocrine sensitivity and chemotherapy resistance in estrogen receptor-positive breast cancer Clin Cancer Res 2007 13 2061 2067 10.1158/1078-0432.CCR-06-2078 17404087

[B21] Pentheroudakis G Kalogeras KT Wirtz RM Grimani I Zografos G Gogas H Stropp U Pectasides D Skarlos D Hennig G Samantas E Bafaloukos D Papakostas P Kalofonos HP Pavlidis N Fountzilas G Gene expression of estrogen receptor, progesterone receptor and microtubule-associated protein Tau in high-risk early breast cancer: a quest for molecular predictors of treatment benefit in the context of a Hellenic Cooperative Oncology Group trial Breast Cancer Res Treat 2008 116 131 143 10.1007/s10549-008-0144-9 18668363

[B22] Frasor J Stossi F Danes JM Komm B Lyttle CR Katzenellenbogen BS Selective estrogen receptor modulators: discrimination of agonistic versus antagonistic activities by gene expression profiling in breast cancer cells Cancer Res 2004 64 1522 1533 10.1158/0008-5472.CAN-03-3326 14973112

[B23] Osborne CK Wakeling A Nicholson RI Fulvestrant: an oestrogen receptor antagonist with a novel mechanism of action Br J Cancer 2004 90 S2 6 10.1038/sj.bjc.6601629 15094757 PMC2750773

[B24] Sui M Huang Y Park BH Davidson NE Fan W Estrogen receptor alpha mediates breast cancer cell resistance to paclitaxel through inhibition of apoptotic cell death Cancer Res 2007 67 5337 5344 10.1158/0008-5472.CAN-06-4582 17545614

[B25] Hug V Hortobagyi GN Drewinko B Finders M Tamoxifen-citrate counteracts the antitumor effects of cytotoxic drugs *in vitro* J Clin Oncol 1985 3 1672 1677 4067614 10.1200/JCO.1985.3.12.1672

[B26] Woods KE Randolph JK Gewirtz DA Antagonism between tamoxifen and doxorubicin in the MCF-7 human breast tumor cell line Biochem Pharmacol 1994 47 1449 1452 10.1016/0006-2952(94)90346-8 8185652

[B27] Albain KS Barlow WE Ravdin PM Farrar WB Burton GV Ketchel SJ Cobau CD Levine EG Ingle JN Pritchard KI Lichter AS Schneider DJ Abeloff MD Henderson IC Muss HB Green SJ Lew D Livingston RB Martino S Osborne CK Adjuvant chemotherapy and timing of tamoxifen in postmenopausal patients with endocrine-responsive, node-positive breast cancer: a phase 3, open-label, randomised controlled trial Lancet 2009 374 2055 2063 10.1016/S0140-6736(09)61523-3 20004966 PMC3140679

[B28] Pico C Martin M Jara C Barnadas A Pelegri A Balil A Camps C Frau A Rodriguez-Lescure A Lopez-Vega JM De La Haba J Tres A Alvarez I Alba E Arcusa A Oltra A Batista N Checa T Perez-Carrion R Curto J GEICAM Group Epirubicin-cyclophosphamide adjuvant chemotherapy plus tamoxifen administered concurrently versus sequentially: randomized phase III trial in postmenopausal node-positive breast cancer patients. A GEICAM 9401 study Ann Oncol 2004 15 79 87 10.1093/annonc/mdh016 14679124

[B29] Sertoli MR Pronzato P Venturini M Mastro LD Queirolo P Vecchio S Taveggia P Campora E Monzeglio C Pastorino S Rubagotti A Bruzzi P Rosso R A randomized study of concurrent versus sequential adjuvant chemotherapy and tamoxifen in Stage II breast cancer [abstract] Proc Am Soc Clin Oncol 2002 21 182

[B30] Goldhirsch A Glick JH Gelber RD Coates AS Thürlimann B Senn HJ Panel members Meeting highlights: international expert consensus on the primary therapy of early breast cancer 2005 Ann Oncol 2005 16 1569 1583 10.1093/annonc/mdi326 16148022

[B31] Kawasaki K Watanabe M Sakaguchi M Ogasawara Y Ochiai K Nasu Y Doihara H Kashiwakura Y Huh NH Kumon H Date H REIC/Dkk-3 overexpression downregulates P-glycoprotein in multidrug-resistant MCF7/ADR cells and induces apoptosis in breast cancer Cancer Gene Ther 2009 16 65 72 10.1038/cgt.2008.58 18654608

[B32] Taira N Doihara H Oota T Hara F Shien T Takahashi H Yoshitomi S Ishibe Y Shimizu N Gefitinib, an epidermal growth factor receptor blockade agent, shows additional or synergistic effects on the radiosensitivity of esophageal cancer cells *in vitro* Acta Med Okayama 2006 60 25 34 16508686 10.18926/AMO/30755

[B33] Takabatake D Fujita T Shien T Kawasaki K Taira N Yoshitomi S Takahashi H Ishibe Y Ogasawara Y Doihara H Tumor inhibitory effect of gefitinib (ZD 1839 Iressa) and taxane combination therapy in EGFR-overexpressing breast cancer cell lines (MCF7/ADR, MDA-MB-231) Int J Cancer 2007 120 181 188 10.1002/ijc.22187 17036319

[B34] Tassone P Tagliaferri P Perricelli A Blotta S Quaresima B Martelli ML Goel A Barbieri V Costanzo F Boland CR Venuta S BRCA1 expression modulates chemosensitivity of BRCA1-defective HCC1937 human breast cancer cells Br J Cancer 2003 88 1285 1291 10.1038/sj.bjc.6600859 12698198 PMC2747554

[B35] Rody A Karn T Gätje R Ahr A Solbach C Kourtis K Munnes M Loibl S Kissler S Ruckhäberle E Holtrich U von Minckwitz G Kaufmann M Gene expression profiling of breast cancer patients treated with docetaxel, doxorubicin, and cyclophosphamide within the GEPARTRIO trial: HER-2, but not topoisomerase II alpha and microtubule-associated protein tau, is highly predictive of tumor response Breast 2007 16 86 93 10.1016/j.breast.2006.06.008 17010609

[B36] Pusztai L Jeong JH Gong Y Ross JS Kim C Paik S Rouzier R Andre F Hortobagyi GN Wolmark N Symmans WF Evaluation of microtubule-associated protein-tau expression as a prognostic and predictive marker in the NSABP-B 28 randomized clinical trial J Clin Oncol 2009 27 4287 4292 10.1200/JCO.2008.21.6887 19667268 PMC2744271

[B37] Jimeno A Hallur G Chan A Zhang X Cusatis G Chan F Shah P Chen R Hamel E Garrett-Mayer E Khan S Hidalgo M Development of two novel benzoylphenylurea sulfur analogues and evidence that the microtubule-associated protein tau is predictive of their activity in pancreatic cancer Mol Cancer Ther 2007 6 1509 1516 10.1158/1535-7163.MCT-06-0592 17483439

[B38] Li JJ Weroha SJ Lingle WL Papa D Salisbury JL Li SA Estrogen mediates Aurora-A overexpression, centrosome amplification, chromosomal instability, and breast cancer in female ACI rats Proc Natl Acad Sci USA 2004 101 18123 18128 10.1073/pnas.0408273101 15601761 PMC539804

[B39] Burow ME Weldon CB Tang Y McLachlan JA Beckman BS Oestrogen-mediated suppression of tumour necrosis factor alpha-induced apoptosis in MCF-7 cells: subversion of Bcl-2 by anti-oestrogens J Steroid Biochem Mol Biol 2001 78 409 418 10.1016/S0960-0760(01)00117-0 11738551

[B40] Tabuchi Y Matsuoka J Gunduz M Imada T Ono R Ito M Motoki T Yamatsuji T Shirakawa Y Takaoka M Haisa M Tanaka N Kurebayashi J Jordan VC Naomoto Y Resistance to paclitaxel therapy is related with Bcl-2 expression through an estrogen receptor mediated pathway in breast cancer Int J Oncol 2009 34 313 319 19148464

[B41] Lien EA Lønning PE Selective oestrogen receptor modifiers (SERMs) and breast cancer therapy Cancer Treat Rev 2000 26 205 227 10.1053/ctrv.1999.0162 10814562

[B42] Obrero M Yu DV Shapiro DJ Estrogen receptor-dependent and estrogen receptor-independent pathways for tamoxifen and 4-hydroxytamoxifen-induced programmed cell death J Biol Chem 2002 277 45695 45703 10.1074/jbc.M208092200 12244117

[B43] Kisanga ER Gjerde J Guerrieri-Gonzaga A Pigatto F Pesci-Feltri A Robertson C Serrano D Pelosi G Decensi A Lien EA Tamoxifen and metabolite concentrations in serum and breast cancer tissue during three dose regimens in a randomized preoperative trial Clin Cancer Res 2004 10 2336 2343 10.1158/1078-0432.CCR-03-0538 15073109

